# Frozen storage of mAbs at elevated temperatures: Balancing stability and sustainability

**DOI:** 10.1016/j.ijpx.2026.100616

**Published:** 2026-07-17

**Authors:** Ricarda Nagel, Nadine Baumeister, Astrid Hauptmann, Reinhard Tober, Karoline Bechtold-Peters, Wolfgang Friess

**Affiliations:** a Pharmaceutical Technology and Biopharmaceutics, Department of Pharmacy, Ludwig-Maximilians-Universität München, Butenandstraße 5-13, 81377 Munich, Germany; b Technical Research and Development, Novartis Pharma AG, 4002 Basel, Switzerland

**Keywords:** Frozen storage, Monoclonal antibodies, Drug substance, Glass transition temperature, Physical stability, Freeze-thaw, Cryoprotection

## Abstract

Protein bulk drug substance (DS) is conventionally kept at −70 to −80 °C to reduce risks such as microbial growth, agitation-related stress, and degradation. This study investigates the long-term physical stability of four IgG-type monoclonal antibodies (mAbs) at −70 and −40 °C under unformulated and fully formulated conditions to probe the broader applicability of −40 °C storage across different antibody formats. For one representative mAb, we further screened minimal formulations and assessed stability at −10 °C. Across all mAbs, −40 °C preserved physical stability comparably to −70 °C. Minimal excipient formulations enabled reliable preservation even above Tg’ at −10 °C. The glass transition temperature (Tg’) serves as a useful guideline, but the difference between the storage temperature and Tg’ is not predictive of stability. Our findings support the adoption of higher frozen-storage temperatures and help guide the optimization of formulations for frozen storage. With this, the study lays the foundation for sustainability, reducing energy consumption without compromising DS quality.

## Highlights

• −40 °C frozen storage is applicable across multiple mAbs and formulation states.
• Tg' defines a stability threshold, while formulation governs frozen-state stability.
• Minimal excipient formulations enable controlled stability above Tg’.
• HPβCD limits particle formation comparably to polysorbate 80 at elevated frozen temperatures.

## Graphical abstract

**Figure:**
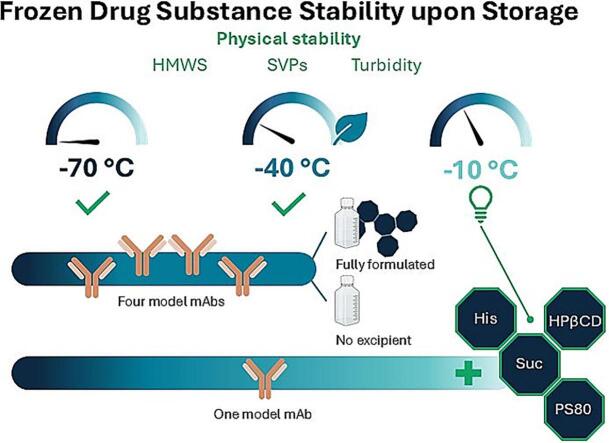

## 1 Introduction

Protein drugs, including monoclonal antibodies, account for a significant portion of global therapies, representing more than 30% of the top 50 products by reported sales in 2024. To preserve their sensitive structures and extend shelf-life, protein bulk drug substance (DS) is typically stored at ultra-low temperatures ranging below −70 to −80 °C, often in large quantities. This minimizes the risks of microbial growth, foaming, agitation during transport, and protein degradation. (Authelin et al., 2020; Bluemel et al., 2020; Rodrigues et al., 2011) Additionally, the intermediate ultra-low-temperature storage enables greater operational flexibility in manufacturing by decoupling DS processing from drug product (DP) processing.

Environmental considerations have become increasingly important in the pharmaceutical industry. With this comes the strive for more sustainable processes. Ultra-low-temperature storage consumes a large amount of energy and adds complexity and demands related to freezing, storage, and thawing. Maintaining the cold chain also presents a logistical transportation challenge. Therefore, ultra-low-temperature storage of DS not only increases environmental impact but also raises operational costs compared to higher-temperature frozen storage. A compromise needs to be found between sustainability and protein stability.

Freezing and frozen storage lower overall reaction rates by the separation of water as ice and reduced matrix mobility through increased viscosity, thereby enhancing both physical and chemical protein stability. Yet, these processes also introduce stress on the protein. Cold denaturation can occur as the free energy of unfolding decreases at lower temperatures. (Privalov, 1990) Additionally, proteins may adsorb and unfold at the formed ice interface. The exposure of aggregation-prone residues upon unfolding may be followed by irreversible aggregation. (Strambini and Gabellieri, 1996; Chang et al., 1996a; Schwegman et al., 2009; Rosa et al., 2017; Arsiccio and Pisano, 2020; Gomes et al., 2021) As water crystallizes, the liquid and non-crystallizing solutes are upconcentrated into a freeze-concentrated matrix (FCM), causing an increase in ionic strength, a redistribution of solutes, and potentially phase separation. (Authelin et al., 2020; Dong et al., 2009; Lehermayr et al., 2011; Hauptmann et al., 2018; Bluemel et al., 2021a; Bluemel et al., 2021b) This may deprive the protein of excipient stabilization. In addition, the buffer pH can shift with temperature due to changes in the dissociation constant and, in extreme cases, due to crystallization of one of the buffering species with lower solubility. (Thorat et al., 2020; Kolhe et al., 2010) Furthermore, the enhanced solubility of oxygen at low temperatures could trigger protein oxidation. (Singh et al., 2009)

To reduce the impact of these stresses, frozen storage strategies commonly rely on maintaining conditions below Tg’ of the FCM. The protein molecules become vitrified in the amorphous matrix, with significantly limited molecular mobility. (Authelin et al., 2020; Kasper and Friess, 2011; Grasmeijer et al., 2013; Thakral et al., 2021) Following the Gordon-Taylor equation, Tg’ depends on the components individual Tg’ and weight fraction, and is independent of the overall concentration. (Kasper and Friess, 2011; Gordon and Taylor, 1952) For pure disaccharide solutions, Tg’ values are typically reported around −30 to −34 °C. (Bluemel et al., 2021a; Whelan et al., 2008; Pansare and Patel, 2016) When combined with protein as a major component with a Tg’ ranging from −10 to −20 °C, Tg’ is increased.

Previous research has mainly examined frozen storage at either ultra-low temperatures below Tg’, such as −80 °C, or at higher temperatures above Tg’, such as −20 and −10 °C. Miller et al. showed that rapid freezing allows protein solutions stored at −20 °C to maintain stability comparable to -80 °C for 6 months, while Bluemel et al. indicated that cryoconcentration shifts in large bulk drug substance are drivers for aggregation and particle formation during storage above Tg’ at −20 °C. Together, these findings demonstrate that stability upon storage above Tg’ can still be maintained, but strongly depends on the freezing method and formulation composition. However, the existing reports are mostly isolated case studies, offering limited generalizability. To this point, there are no detailed studies assessing long-term stability at intermediate temperatures below Tg’. For many protein DS formulation compositions, the Tg’ is above −40 °C. This temperature thus represents a more sustainable and stability-providing storage condition alternative to −70 or −80 °C. From a practical perspective, −40 °C is the lowest temperature achievable with single compressor freezers, whereas lower temperatures require cascade refrigeration using two compressors. This is associated with substantially higher energy consumption, capital cost, and maintenance complexity, making −40 °C a particularly attractive setpoint when balancing stability with sustainability.

To address this gap, we investigate two complementary aspects, both focusing on DS, where long-term frozen storage is most relevant. DP formulations, which are typically stored under refrigerated conditions, are not within the scope of this study. We show that −40 and −70 °C are equivalent long-term storage temperatures for four different IgG1-type mAbs, both in excipient-free and fully formulated DS states. The four mAbs differ in their primary sequences, isoelectric points, and individual degradation liabilities, representing a structurally diverse IgG1 panel and thereby providing a broad basis for the applicability of −40 °C as a sustainable storage temperature. The full DS formulation is expected to ensure optimal protection against stresses during freezing, frozen storage, and thawing, and simplifies subsequent DP fill and finish operations. A minimal-excipient or completely excipient-free approach offers flexibility, particularly during clinical development, and may also provide good protection. In this context, the minimal-excipient strategy was deliberately chosen to maintain comparatively high Tg' values while still retaining sufficient excipient-mediated stabilization. This should provide a balance between matrix immobilization and cryoprotective effects on the one hand and enabling storage at −40 °C below Tg' on the other hand. As Tg' of the FCM is determined by the mass ratio of its constituents rather than their absolute concentrations, formulations with a higher relative mAb fraction yield higher Tg' values. The mAb-to-excipient ratios in the present study are equal to or shifted towards a higher mAb fraction compared to prior work. Thus the Tg' values of formulations investigated are considered to be equal to or higher, being below Tg’ at both −70 and −40 °C (Bluemel et al., 2021a; Nagel et al., 2026) With complete removal of excipients, Tg’ is highest, but the excipient-mediated stabilization in the FCM, which then consists exclusively of mAb, is lacking. As a second aspect, we mechanistically evaluate excipient-mediated stabilization across different minimal DS formulations using an IgG1-type model mAb stored at −70, −40, and −10 °C. MAb 1 was selected as the model mAb for this arm based on prior internal experience indicating greater sensitivity to freeze-thaw stress compared to the other mAbs in the panel. This makes mAb 1 a suitable stress model to amplify and resolve formulation-dependent differences in stability. Here, −10 °C was included as a stress model above Tg’, as it is more sensitive to formulation effects and represents a potential shorter-term option for frozen storage. Histidine buffer is added to assess the impact of charge stabilization and ionic strength, as it remains amorphous during freezing and does not show pronounced pH shifts. (Kolhe et al., 2010) Evidence from molecular dynamics simulations also suggests that histidine can temporarily adsorb to hydrophobic regions on the protein surface, preventing aggregation. (Saurabh et al., 2022) Sucrose was tested as a classical cryoprotectant stabilizing via preferential hydration and matrix vitrification. (Arakawa and Timasheff, 1982; Arakawa et al., 2001; Bhatnagar et al., 2008; Arsiccio and Pisano, 2017) Sucrose concentrations from 3 to 200 mM were investigated, with 200 mM reflecting typical industry practice for combined cryoprotection and osmolality adjustment to isotonicity. (2-Hydroxypropyl)-β-cyclodextrin (HPβCD), a cyclic polyol, was included as a mechanistic comparator to sucrose, offering higher Tg’ and being known to stabilize against interface-induced degradation. Finally, polysorbate 80 (PS80) was included as a benchmark interfacial stabilizer. The two study arms provide both mechanistic and practical guidance for optimizing frozen-storage strategies.

## 2 Materials and methods

### 2.1 Materials

Novartis AG (Basel, Switzerland) supplied different stock formulations of all model mAbs used in this study.

L-histidine, L-histidine monochloride monohydrate, 0.1 M HCl, 0.1 M NaOH, and sucrose were obtained from Merck KGaA (Darmstadt, Germany). HPβCD was supplied by Wacker Chemie AG (Burghausen, Germany). Sodium sulfate, potassium dihydrogen phosphate, dipotassium hydrogen phosphate, sodium chloride, and glacial acetic acid were purchased from VWR.

Uncoated 6R glass vials were purchased from SCHOTT AG (Mainz, Germany) and stoppered with FluroTec® lyophilization stoppers from West Pharmaceuticals (Eschweiler, Germany).

### 2.2 Sample preparation

Fully formulated mAb solutions were prepared by diluting the stock solutions to 10 mg/mL with the appropriate formulation. To remove surfactant, when present, and other excipients from the DS formulation, the protein was precipitated by adding a saturated sodium sulfate solution. The suspensions were filtered through a 0.2 μm bottle-top filter and washed at least five times with the concentrated sodium sulfate solution to remove all traces of excipients. The mAbs were then redissolved with highly purified water (HPW) and dialyzed against HPW using Slide-A-Lyzer® cassettes (Thermo Fisher Scientific Inc., Waltham, Massachusetts, USA) with a 30 kDa MWCO PES membrane. The absence of surfactant was confirmed by HPLC-CAD. The stock solutions were diluted with HPW. The pH was confirmed at 6.0 and, if necessary, adjusted by adding 0.1 M HCl or NaOH, before final dilution to 10 mg/mL.

For excipient evaluation, only mAb 1 was used as the model mAb, as listed in Table 1. For formulations containing histidine buffer, the mAb stock was dialyzed against 10 mM histidine buffer. Samples containing sugars were prepared by dissolving the appropriate amount of sugar in a defined volume of mAb stock and adjusting the final mAb concentration. PS 80 was introduced by spiking a PS80 stock solution. The mAb concentration was determined via UV absorbance at 280 nm using a NanoDrop 2000 (Thermo Fisher Scientific, Waltham, MA, USA). Before filling, all formulations were steril filtered using 0.2 μm PVDF membrane syringe filters (Acrodisc®, Pall GmbH, Dreieich, Germany). Samples were prepared in triplicate for each formulation and time point by filling 3 mL into 6R glass vials. The vials were stoppered and crimped before freezing.

**Table 1:** Formulations tested with 10 mg/mL mAb 1.

Excipients [mM]			
Histidine	Sucrose	HPβCD	PS80
–	–	–	–
–	200	–	–
–	25	–	–
–	13	–	–
–	3	–	–
–	200	6.84	–
–	–	6.84	–
–	–	3.42	–
–	–	0.34	–
–	200	–	0.3
10	200	6.84	–
10	200	–	–
10	–	–	–

The samples were frozen in an MKF240 air-blast climate chamber (Binder GmbH, Tuttlingen, Germany). After equilibration at 20 °C for 1 h, the samples were cooled from 20 to −40 °C at a rate of 10 °C/min, and the temperature was then maintained at −40 °C for 16 h to ensure complete freezing.

Samples were transferred to freezers at −80 °C (LAUDA-GFL GmbH, Burgwedel, Germany), −40 °C (Liebherr, Bulle, Switzerland), and −10 °C (Köttermann GmbH, Uetze, Germany) for long-term storage. The study comprised two arms. In the first arm, the four mAbs in non-formulated and fully formulated DS were stored at −70 and −40 °C. Samples were assessed at liquid t0 (prior to freezing), frozen t0 (after 24 h of equilibration at the storage temperature), and after 12 months of storage. In the second arm, mAb 1 formulations were stored at −70, −40, and −10 °C and assessed at the same timepoints. Triplicate samples of each formulation were thawed on the laboratory bench at 20 ± 2 °C prior to analysis.

### 2.3 Size-exclusion chromatography

Size-Exclusion Chromatography (SEC) was performed using an Agilent 1200 series HPLC system with UV/Vis detection at 220 nm (Agilent Technologies, Santa Clara, CA, USA), a TSKgel SWxl column (Tosoh Bioscience GmbH, Griesheim, Germany), and 150 mM potassium phosphate buffer pH 6.5, at 0.4 mL/min. Samples were centrifuged for 3 min at 17,000 x g using a Micro Star 17R (VWR International GmbH, Darmstadt, Germany), and 4 μL of the supernatant were injected. The content of higher molecular weight species (HMWS) was quantified using Agilent OpenLAB Data Analysis Software 2.1.

### 2.4 Flow imaging microscopy

Subvisible particles (SVPs) were measured using a FlowCam 8100 (Fluid Imaging Technologies, Scarborough, Maine, USA) with a 10× magnification cell. Measurements were processed with VisualSpreadsheet® 4.7.6 software. Particles were counted in a 160 μL sample, with a flow rate of 0.15 mL/min. The auto image frame rate was 28 frames/s, sampling for 60 s. Particle identification used a nearest neighbor threshold of 3 μm, with segmentation thresholds set at 10 for light pixels and 13 for dark pixels.

### 2.5 Turbidity

Sample turbidity was measured with 1.8 mL samples according to Ph. Eur. 2.2.1 using a Hach TL2360 Turbidimeter (Hach Lange GmbH, Düsseldorf, Germany), operating in nephelometric mode with detection of scattered light at a 90° angle. The instrument was calibrated with formazine reference standards covering the region of interest, and data are reported in formazine turbidity units (ftu). Additionally, all vials were visually evaluated for visible particles under ambient laboratory lighting.

## 3 Results

### 3.1 High molecular weight species (HMWS)

High-molecular-weight species, such as mAb dimers and trimers, are often used as a gold standard indicator of mAb stability and are sensitive to early signs of aggregation. We compared HMWS levels over time for four different mAbs in unformulated and in full DS formulations, and for one model mAb (Table 1) in different minimal formulations, to assess the impact of the different frozen storage temperatures on stability.

**Fig. 1:**
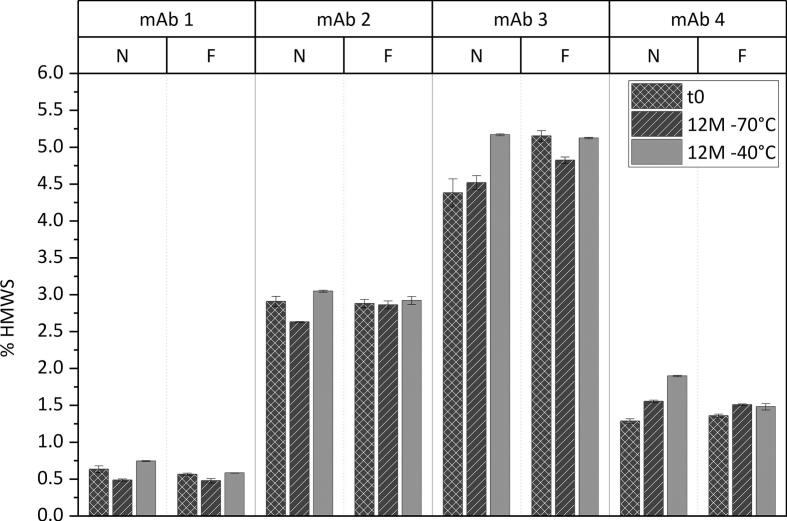
%HMWS upon 12-month storage at −70 and −40 °C for four different mAbs as non- or fully formulated DS.

For mAb 1, the initial HMWS levels were 0.6%, while for mAb 2, the initial levels were 2.9% for the unformulated and fully formulated DS, respectively (Fig. 1). In both cases, the HMWS level remained largely unchanged for 12 months, regardless of storage at −70 or −40 °C. For mAb 3, the initial HMWS levels were 4.4% for the unformulated and 5.2% for the fully formulated DS. During storage, HMWS levels for the unformulated DS at −70 °C remained constant, while at −40 °C an increase to 5.2% was observed. For the fully formulated DS at −70 °C, a marginal decrease to 4.8% was detected, while HMWS levels at −40 °C did not change. For mAb 4, the initial HMWS levels were 1.3% and 1.4%, respectively, for the unformulated and fully formulated DS. Upon storage, HMWS levels in the unformulated DS increased slightly to 1.6% at −70 °C and to 1.9% at −40 °C, whereas in the fully formulated DS, the values remained within ±0.1% of the starting value. In summary, small changes in HMWS levels are observed upon storage in two of the four unformulated mAbs. This trend was evident at both storage temperatures, −70 °C and − 40 °C, but appeared more pronounced at −40 °C.

**Fig. 2:**
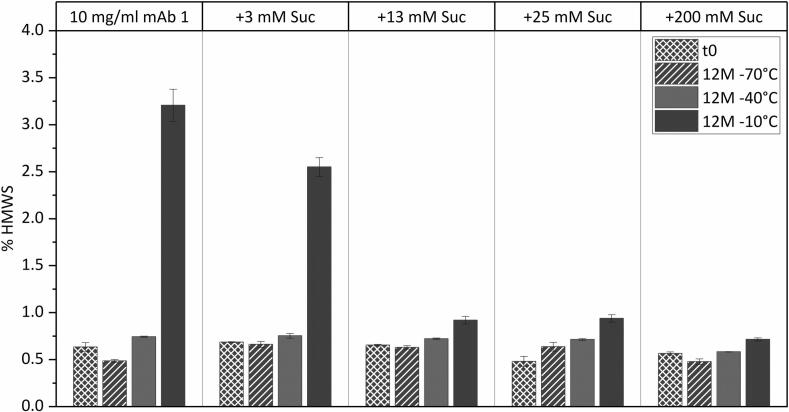
%HMWS upon 12-month storage at −70, −40, and −10 °C for mAb1 in combination with different sucrose concentrations.

**Fig. 3:**
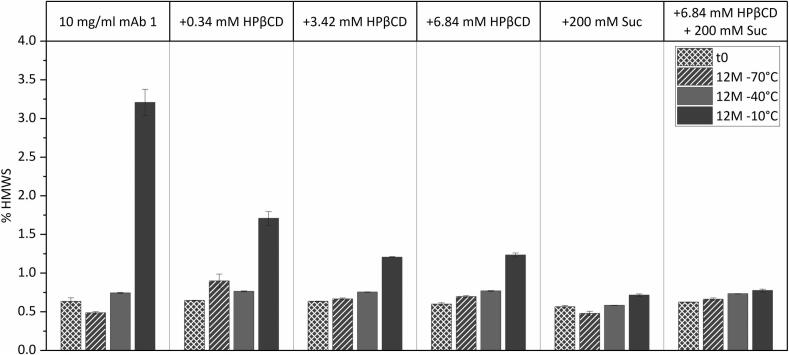
%HMWS upon 12-month storage at −70, −40, and −10 °C for mAb1 in combination with different HPβCD concentrations.

Subsequently, mAb 1 was investigated in various minimal formulations. For pure mAb, the HMWS level remains at 0.5% and 0.7%, within ±0.1% to t0, upon storage at −70 and −40 °C. In formulations containing sucrose, equivalent good stabilization at both −70 and −40 °C was observed (Fig. 2). In contrast to −40 °C, for unformulated mAb at −10 °C, a pronounced increase of HMWS levels to 0.6% to 3.2% was observed. With increasing sucrose concentration, HMWS formation notably decreases. At −10 °C, the formulation containing 3 mM sucrose showed a pronounced increase in HMWS levels, from 0.7% to 2.6%. At 13 mM sucrose, this was limited to an increase to 0.9%. At 200 mM, HMWS levels show only a minor variation within ±0.1%. Thus, only a slight additional increase in stability is achieved by increasing the sucrose concentration further. For HPβCD formulations (Fig. 3), HMWS levels also showed a concentration-dependent relationship. The HMWS levels of the formulation containing 0.34 mM HPβCD increased from 0.7% to 1.7% upon storage at −10 °C. At 3.42 mM HPβCD, HMWS levels increased only to 1.2%. More HPβCD (6.84 mM) did not result in even lower levels. In the combination of 6.84 mM HPβCD with 200 mM sucrose, the HMWS level rises from an initial 0.6% to 0.8%, similar to the values recorded for 200 mM sucrose alone.

**Fig. 4:**
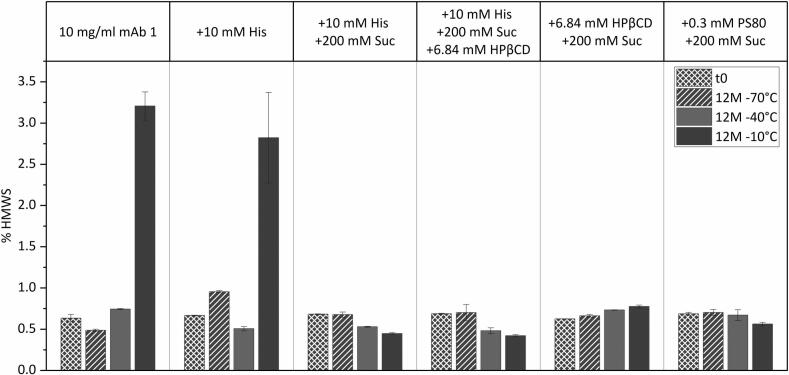
%HMWS upon 12-month storage at −70, −40, and −10 °C for mAb1 in combination with buffer, sugar, and interfacial stabilizer.

Upon addition of 10 mM histidine to the pure mAb (Fig. 4), HMWS levels increase from 0.7% at t0 to 2.8% upon storage. Here, adding 200 mM sucrose to histidine reduced HMWS levels to 0.4%. Adding 6.84 mM HPβCD yields values similar to those for the combination of 200 mM sucrose and 6.84 mM HPβCD without histidine. If HPβCD is replaced with 0.3 mM PS80 in combination with sucrose, HMWS levels remain at their initial value of 0.7%.

### 3.2 Subvisible particles (SVPs)

**Fig. 5:**
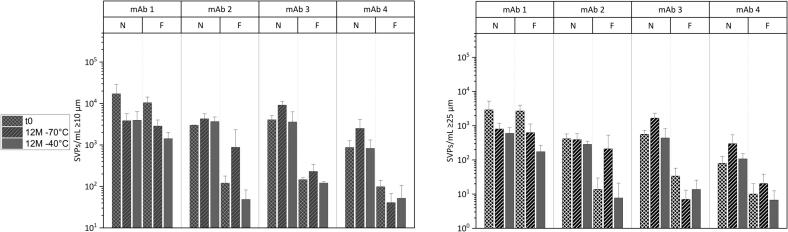
SVPs upon 12-month storage at −70 and −40 °C for four different mAbs as non- and fully formulated DS.

We also assessed larger aggregate formation by SVP analysis. Prior to sampling, visual evaluation of vials under ambient laboratory light was carried out, confirming the absence of visible particles in all samples. Regarding SVPs, similar counts, both low and close to t0 values, can be observed for the different antibodies, regardless of whether they are stored at −70 or −40 °C (Fig. 5). In contrast to HMWS levels, which were comparable between non-formulated and fully formulated DS, SVP counts were lower for the fully formulated DS than for the non-formulated DS.

**Fig. 6:**
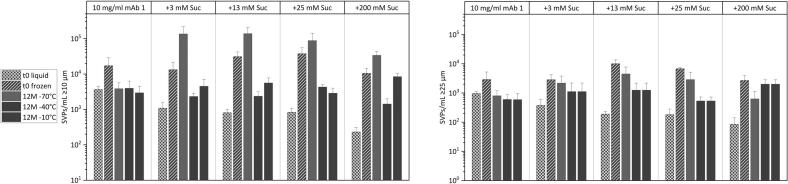
SVPs upon 12-month storage at −70, −40, and −10 °C for mAb1 in combination with different sucrose concentrations.

**Fig. 7:**
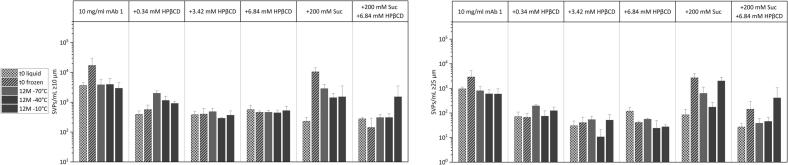
SVPs upon 12-month storage at −70, −40, and −10 °C for mAb1 in combination with different HPbetaCD concentrations.

**Fig. 8:**
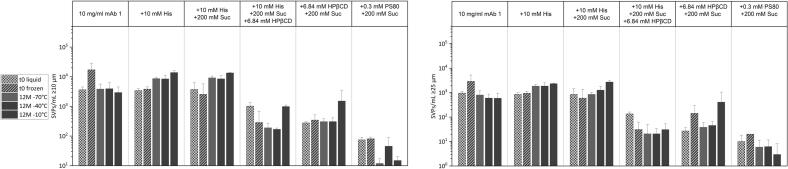
SVPs upon 12-month storage at −70, −40, and −10 °C for mAb1 in combination with buffer, sugar, and interfacial stabilizer.

In the SVP analysis of mAb 1 formulations, two t0 values are reported: one for the liquid sample prior to freezing and thawing, and one for samples frozen and thawed after 24 h of equilibration (Section 2.2). All liquid t0 samples show consistently low particle counts across formulations. For sucrose containing samples at frozen t0, SVP counts are increased, while they remain lower for other formulations (Fig. 6). This selective increase is not reflected in HMWS levels, which remained low across all sucrose concentrations and storage temperatures. After 12 months of storage, SVP counts are below the t0 frozen values and closer to t0 liquid, although still slightly elevated. Counts at −70 °C are generally higher than those at −40 and −10 °C. In formulations containing HPβCD, SVP counts remain unchanged at all storage temperatures, including −10 °C, throughout the 12-month study (Fig. 7). The counts of HPβCD-containing formulations are markedly lower than those for non-formulated mAbs and formulations with sucrose, with no concentration-dependent differences in SVPs across the tested HPβCD concentrations. In contrast, HMWS levels showed concentration-dependent increases at −10 °C, with lower HPβCD concentrations yielding higher HMWS. No synergistic effect on SVPs was observed for a combination of 200 mM sucrose and 6.84 mM HPβCD. Upon addition of 10 mM histidine to the non-formulated mAb, slightly elevated SVP counts at t0 were observed, with counts being similar for −70 and −40 °C stored samples (Fig. 8). A slight trend towards more SVPs for samples stored at −10 °C was observed, in line with the higher HMWS levels for this formulation. Combining 10 mM histidine with 200 mM sucrose does not alter this outcome. When an additional 6.84 mM HPβCD is added, the SVP counts are notably reduced at t0 frozen and stay at that level, regardless of storage conditions. Compared to the formulation with only 200 mM sucrose and 6.84 mM HPβCD, with no histidine, there was no difference in SVP counts. A combination of 200 mM sucrose and 0.3 mM PS80, a standard interfacial stabilizer, yielded even lower SVP counts. Nevertheless, the difference in SVP counts between 200 mM sucrose with either 6.84 mM HPβCD or 0.3 mM PS80 is only minor.

### 3.3 Turbidity

**Fig. 9:**
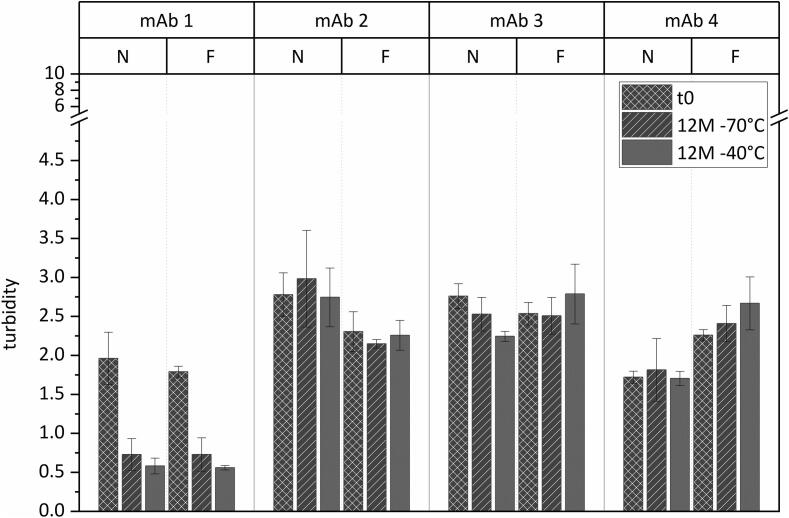
Turbidity (ftu) upon 12-month storage at −70 and −40 °C for four different mAbs as non- and fully formulated DS.

Turbidity was measured as an additional parameter because it reflects changes in particle levels across a wide range of sizes. For the different mAbs in both non-formulated and fully formulated DS, absolute turbidity values varied between individual mAbs and between non-formulated and formulated states, without following the trends observed for HMWS or SVP counts. Differences appeared as fluctuations rather than consistent trends, and turbidity did not change significantly during freezing, thawing, or 12 months of storage at either −70 or −40 °C (Fig. 9).

**Fig. 10:**
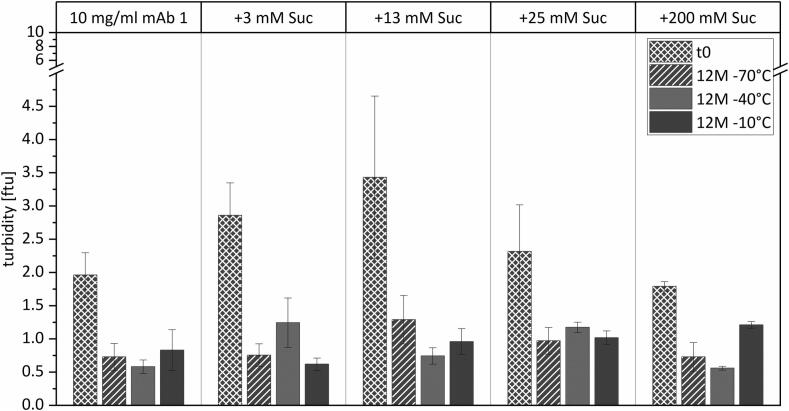
Turbidity (ftu) upon 12-month storage at −70, −40, and −10 °C for mAb1 in combination with different sucrose concentrations.

**Fig. 11:**
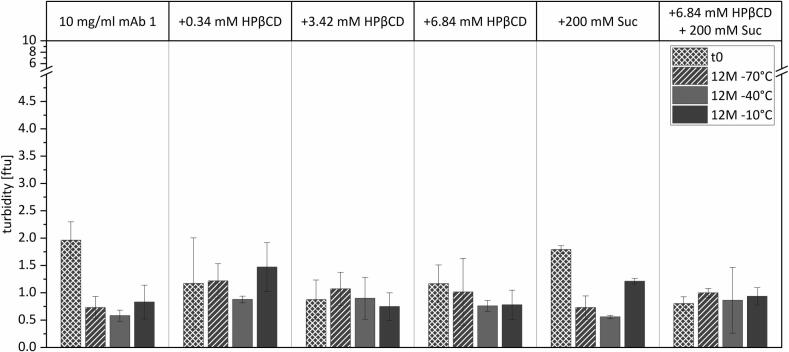
Turbidity (ftu) upon 12-month storage at −70, −40, and −10 °C for mAb1 in combination with different HPβCD concentrations.

For mAb 1 formulations, liquid t0 turbidity values were low between 0.6 and 0.8 ftu for most formulations. The higher SVP counts for sucrose formulations observed at frozen t0 were mirrored by a higher turbidity (Fig. 10), while HMWS levels remained low. The turbidity of all samples stored at −70, −40, or −10 °C for 12 months remained low, falling back close to the liquid t0 baseline, and did not show a consistent increase beyond this value at any sucrose concentration. For formulations containing any concentration of HPβCD, the results were similar, except for the higher t0 values observed in frozen and thawed samples compared to those before freezing and thawing. (See Fig. 11.)

**Fig. 12:**
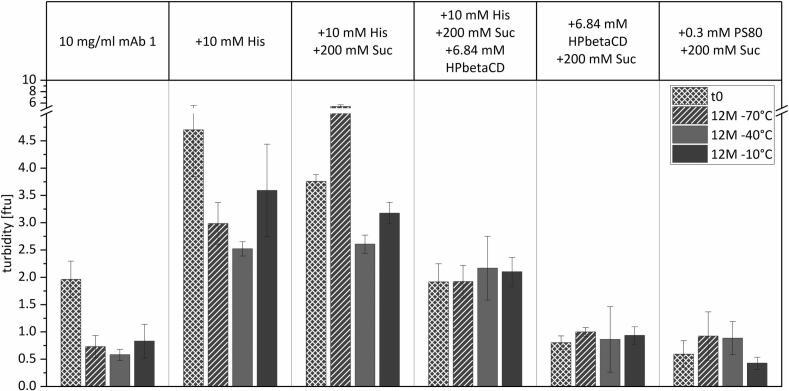
Turbidity (ftu) upon 12-month storage at −70, −40, and −10 °C for mAb1 in combination with buffer, sugar, and interfacial stabilizer.

In comparison to non-formulated mAb, the addition of 10 mM histidine leads to overall higher turbidity at liquid t0 (2.7 ftu), independent of the presence of 200 mM sucrose (Fig. 12), comparable to the elevated SVP counts for histidine-containing formulations. In contrast, HMWS levels for histidine +200 mM sucrose were lower (0.4%) than for histidine alone. Addition of 6.84 mM HPβCD to this combination slightly reduced turbidity (2.0 ftu), and the combination of only 200 mM sucrose and 6.84 mM HPβCD without histidine yielded clearly lower values, comparable to 200 mM sucrose with 0.3 mM PS80. These differences in liquid t0 did not change significantly during storage at −70, −40, or −10 °C. For none of the formulations containing HPβCD only, or a combination of buffer, sugars, and interfacial stabilizer, a consistent and progressive change of turbidity during the 12 months of storage at any storage temperature was observed.

## 4 Discussion

This study systematically evaluates the long-term physical stability of four mAbs during frozen storage under different temperature conditions and formulations. We compare three temperature settings: standard ultra-low (−70 °C), a more sustainable option (−40 °C), and an elevated option (−10 °C). Additionally, we assess different formulations at various concentrations and combinations, focusing on their ability to maintain physical stability for one of the mAbs. For the formulation comparison, our approach emphasizes practical guidance by identifying the minimum excipient requirements needed for effective stabilization. Our results also provide new insights into the use of Tg' as a threshold value for storage decisions, clarifying its limitations and implications. Overall, findings offer new insights into mAb stability, the protective roles of excipients, mechanisms of aggregation and particle formation, and practical guidance for sustainable storage at higher frozen temperatures.

For fully formulated drug substance, HMWS (high-molecular-weight species), SVPs (subvisible particles), and turbidity levels remain largely unchanged over 12 months at both −70 and −40 °C. This confirms a similar good physical stability despite storage close to Tg’ at −40 °C. Tg’ has been shown to be a critical parameter in frozen storage. Below Tg’, molecular mobility is restricted, which generally favors stability. (Authelin et al., 2020; Kolhe et al., 2010; Franks, 1994; Alhalaweh et al., 2015) Seifert et al. have shown an exponential increase in the viscosity of the FCM towards Tg', which can be directly linked to a decrease in reaction rate. (Seifert and Friess, 2020) However, our data and literature evidence stress that maximal stability below Tg’ cannot be directly assumed. Contrary to a universal stability below Tg’, for two mAbs, slight increases in HMWS were observed even when stored well below Tg’. (Chang et al., 1996b) We chose a completely non-formulated approach to achieve a Tg’ around −18 °C for a higher frozen storage temperature of −40 °C. (Bluemel et al., 2021a) This was hypothesized to result in good stabilization, as the residence time in the mobile FCM during freezing is shortened, while on the other hand, a larger safety margin between storage temperature and Tg’ is provided. (Alhalaweh et al., 2015) Fang et al. have previously demonstrated that increasing the residence time in the mobile FCM, defined as the interval between ice nucleation and reaching Tg’, leads to more aggregation. (Fang et al., 2020) A higher nucleation temperature resulted in a lower specific surface area (SSA) of the ice interface, which correlated with reduced HMWS. For the non-formulated mAbs in this study, the complete absence of excipients and slow cooling at 10 °C/min may have led to a higher nucleation temperature, resulting in a low SSA, while the high Tg’ on the other hand ensures a shorter residence time. This may compensate for the missing stabilizers and help preserve overall physical stability. The observed changes in HMWS are considered minor and do not compromise overall stability.

As described before, sucrose-containing formulations of mAb 1 exhibited higher SVP values at t0 frozen than at t0 liquid, with values closer towards baseline upon 12-month storage. This observation may be explained with the higher variability of freeze-thaw induced particle formation due to the stochastic nature of freeze-thaw processes, and the dominance of freeze-thaw over evolution during storage. Flow imaging microscopy does not allow unambiguous assignment of detected SVPs to protein- or excipient-derived origin. Yet the full solubility and amorphous behavior of the excipients used, together with previous data on the same sucrose and HPβCD materials showing considerably lower SVP counts in protein-free solutions, indicate that the SVPs reflect predominantly protein-related particles. (Nagel et al., 2026) Some stress factors, such as cryoconcentration and the kinetics of aggregation, may also influence outcomes even in a low-mobility environment. (Fang et al., 2020; Takenaka et al., 1996) Takenaka et al. have proposed that, due to cryoconcentration, higher-order reactions in the frozen state may be accelerated despite the sharp decrease in the reaction constant k at lower temperatures. (Takenaka and Bandow, 2007) Protein aggregation follows mostly higher-order kinetics and thus increases exponentially with concentration. (Kendrick et al., 1998) A general trend towards slightly higher SVP counts, especially seen in non-formulated and sucrose-containing samples at lower temperatures, further underscores the protective effect of excipients and the importance of formulation optimization, even below Tg’. The literature contains evidence from both experimental and molecular dynamics-based studies indicating that protein structure can be disrupted during freezing. If the FCM is more mobile, these structures may refold more efficiently, resulting in less aggregation, while remaining in an unfavorable state at lower temperatures, where mobility is decreased.

It is possible that the systematic decrease in SVP counts observed at higher storage temperatures results from increased dissociation of reversible particles, driven by higher mobility. However, this increase followed by a decrease is observed only in formulations containing sucrose, whereas formulations without sucrose do not exhibit this behavior. Conversely, instability may be as effectively countered by the right stabilizers as by solidification, even if the formulation is stored close to or above its Tg’, as seen for the fully formulated mAbs. In summary, the found equivalence of both −70 and −40 °C suggests that the industry-standard ultra-low-temperature storage may not always be necessary to maintain mAb integrity, provided that formulations are optimized.

When the storage temperature was further elevated above Tg’ to −10 °C, the results were more nuanced. For pure mAb 1 and in combination with 10 mM histidine buffer at −10 °C, a pronounced increase in HMWS levels was observed, indicating that physical stability is more sensitive to formulation at this temperature, in line with previous findings. (Singh et al., 2011) However, the SVPs and turbidity at −10 °C remained similar to the values seen at −70 and −40 °C, despite the absence of stabilizers. Already the addition of the least amounts of excipients such as sucrose and HPβCD mitigated the increase in HMWS at −10 °C. For sucrose, low concentrations ≥25 mM were sufficient to protect the protein against physical degradation, with higher concentrations offering only marginal additional benefit. The proposed mechanism of cryoprotection by sugar is based on its preferential exclusion from the protein, and consequently, the preferential hydration of the protein by water molecules. (Lee and Timasheff, 1981; Kendrick et al., 1997) This results in a larger unfolding enthalpy, thereby stabilizing the protein in its native state. However, the concentration required to achieve this stabilizing effect during freezing and within the frozen state is not fully elucidated, as the solutes get upconcentrated in the FCM. At 25 mM sucrose, the volume occupied by the sugar molecules is roughly a fifth of that occupied by the mAb molecules. Therefore, a thermodynamic stabilization appears more likely in this case than embedment in a highly viscous matrix. (Grasmeijer et al., 2013; Chang et al., 2005; Cicerone and Douglas, 2012) Often, sugars are added at higher concentrations in the drug substance formulation to simultaneously achieve cryoprotection for storage and an adjusted osmolality for the final drug product formulation. This is a convenient approach; yet, our results show that, if necessary, already very low concentrations provide sufficient cryoprotection.

HPβCD also demonstrated effective stabilization, in some regards even superior to sucrose. Upon addition of 0.34 mM HPβCD, HMWS levels increased less than with 3 mM sucrose. This likely can be attributed to thermodynamic stabilization through preferential hydration, similar to sugars. Similar to other sugars, HPβCD possesses the ability to bind water, but has a higher binding capacity than sucrose. (Gharsallaoui et al., 2008; Arsiccio et al., 2021) Thus, even lower amounts may lead to the observed stabilization. As for sucrose, this stabilizing effect increases with higher concentrations but reaches a plateau with 3.42 mM HPβCD. Despite the small difference, this could be, on the one hand, due to potentially less efficient cryoprotection by HPβCD. On the other hand, HPβCD has been shown to preferentially stabilize proteins in a partially unfolded state, possibly promoting reversible oligomerization while maintaining overall physical stability. (Huang et al., 2024) Our previous results also show a slight trend towards higher percentages of HMWS in formulations containing HPβCD. Only physical stability was evaluated in both studies, and our analytical panel did not include any potency assays. Therefore, further investigation is needed before drawing relevant conclusions regarding this finding.

Superior stabilization by HPβCD was found regarding the formation of particles. SVP counts remained remarkably low, and no particle formation at all was observed at −10 °C across all concentrations investigated. This distinct difference compared with sucrose formulations suggests stabilization via mechanisms distinct from cryoprotection. The ability of HPβCD to stabilize proteins against physical degradation at air-liquid interfaces is documented in the literature. (Huang et al., 2024; Stolzke et al., 2022; Zhang et al., 2022; Li et al., 2024) Serno et al. investigated the ability of various cyclodextrin derivatives to stabilize against ice-liquid interfacial stresses during freeze-thaw cycling, with 2.5 mM HPβCD sufficient to preserve mAb against degradation. (Serno et al., 2011) Although HPβCD reduces interfacial tension to some extent, it does not displace the protein from the interface as effectively as polysorbates. This is reflected by the even lower SVP counts in the 200 mM sucrose and 0.3 mM PS80 formulation. Nonetheless, the overall particle counts for both formulations remained very low upon storage, indicating that HPβCD can offer effective interfacial stabilization at the ice liquid interface during frozen storage. In this study, no significant benefit of adding 10 mM histidine for physical stability was observed. Small effects, such as slightly decreased HMWS formation, were overshadowed by the more pronounced stabilizing effects of sugars and surfactant. Histidine-containing formulations did show slightly elevated turbidity values at liquid t0 (Section 3.3), which were not accompanied by a corresponding increase in HMWS or SVP counts. This suggests that this effect does not stem from soluble oligomers or micron-sized particles, but more likely from species in between the resolution windows of the two methods. A plausible explanation is the modest increase in ionic strength partially screening electrostatic repulsion between mAb molecules andfavoring weak, reversible self-association. (Bluemel et al., 2021b) Importantly, this effect did not progress during freezing, thawing, or storage, indicating that histidine slightly modulates solution behavior without contributing to freeze-thaw- or storage-induced destabilization. We previously reported that HMWS formation during storage decreased with higher histidine concentrations. Although not assessed in this study, adding a buffer might improve chemical stability.

In summary, we have shown that many formulations provide acceptable stabilization over 12 months of storage, including at −10 °C. For this, only minimal concentrations of stabilizing excipients are needed.

## 5 Conclusion

This study aimed to expand the knowledge space on the impact of long-term frozen storage at

-40 °C on the physical stability of mAbs and to assess its broad applicability in comparison to ultra-low temperature storage at −70 °C. The physical stability was evaluated thoroughly by measuring HMWS, SVPs, and turbidity and by performing a visual evaluation. For a selected mAb model, combinations of different excipients were explored to identify optimal minimal formulations that may enable storage above Tg’ at −10 °C.

Our results demonstrate that equivalent good stabilization is achieved during long-term frozen storage at −40 and −70 °C. These findings applied broadly to the four different tested mAbs under both excipient-free and fully formulated conditions. In addition, our findings show that an optimized formulation can maintain physical stability reliably above Tg’ at −10 °C for up to 12 months. This may serve as the basis for short-term storage or shipping at −10 °C, provided that the excipient type and concentrations are carefully selected. Only minimal amounts of stabilizers, such as sucrose and HPβCD, are needed, and combining excipients may offer synergistic benefits. Interfacial protection was achieved with HPβCD as effectively as with PS80, and particle formation and aggregation were similarly mitigated when combined with sucrose. This makes HPβCD a promising excipient, offering stabilization without the degradation liabilities of polysorbates. Additionally, HPβCD enables simplified downstream processing. Unlike polysorbates, it does not form self-assembling micelles. As a result, it can be conveniently removed during ultrafiltration/diafiltration (UF/DF).

Further studies are needed to assess chemical stability and to advance understanding of how pH affects frozen-state stability. In addition, higher mAb concentrations should be investigated. Higher concentrations have been shown to inherently stabilize proteins against stresses during freezing, but it remains unclear whether some stabilizing mechanisms depend on the stabilizer-to-protein ratio rather than on their absolute concentrations.

In summary, this work shows that four IgG1-type mAbs remain equally stable at −40 and −80 °C over 12 months, independent of formulation. Together with previously published data, these findings broaden the experimental basis supporting the general concept that, if storage occurs below Tg’, the specific temperature is of only marginal relevance to physical stability. While product-specific confirmation remains necessary, given the molecule-specific nature of degradation kinetics, the broad applicability across four structurally distinct mAbs underscores that −40 °C offers a reliable and more energy-efficient alternative, while it may also facilitate more flexible logistics.

## CRediT authorship contribution statement

**Ricarda Nagel:** Writing – review & editing, Writing – original draft, Visualization, Validation, Project administration, Methodology, Investigation, Formal analysis, Data curation, Conceptualization. **Nadine Baumeister:** Investigation, Formal analysis, Data curation. **Astrid Hauptmann:** Supervision. **Reinhard Tober:** Supervision. **Karoline Bechtold-Peters:** Supervision, Resources, Conceptualization. **Wolfgang Friess:** Writing – review & editing, Supervision, Resources, Conceptualization.

## Declaration of competing interest

The authors declare the following financial interests/personal relationships which may be considered as potential competing interests:

Wolfgang Friess reports equipment, drugs, or supplies was provided by Novartis Pharma AG. If there are other authors, they declare that they have no known competing financial interests or personal relationships that could have appeared to influence the work reported in this paper.

## Data Availability

The data that has been used is confidential.

## Appendix A Supplementary data

Supplementary material: Frozen Storage of mAbs at Elevated Temperatures: Balancing Stability and Sustainability. IMAGE 1
