# Supplementary material for: Frozen storage of mAbs at elevated temperatures: Balancing stability and sustainability
Source: Int J Pharm X. 2026 Jul 17;12:100616. doi: 10.1016/j.ijpx.2026.100616 (PMC13393409; doi:10.1016/j.ijpx.2026.100616)
Supplement: Supplementary file 1 — Supplementary material: Frozen Storage of mAbs at Elevated Temperatures: Balancing Stability and Sustainability. [file mmc1.docx]

# **Supplementary Materials**

Frozen Storage of mAbs at Elevated Temperatures: Balancing Stability and Sustainability

Ricarda Nagel^1^, Nadine Baumeister^1^, Astrid Hauptmann^2^, Reinhard Tober^2^, Karoline Bechtold-Peters^2^, Wolfgang Friess^1*^

^1^Pharmaceutical Technology and Biopharmaceutics, Department of Pharmacy, Ludwig-Maximilians-Universität München, Butenandstraße 5-13, 81377 Munich, Germany

^2^ Technical Research and Development, Novartis Pharma AG, 4002 Basel, Switzerland

*Corresponding Author: Wolfgang Friess, wolfgang.friess@lrz.uni-muenchen.de

# High molecular weight species (HMWS) of mAb 1 formulations measured by HP-SEC at t0, and after 3, 6, and 12 months of storage at -70, -40, and -10 °C

|   **Figure S1.** %HMWS in 10 mg/mL mAb1 in combination with different sucrose concentrations |   **Figure S2.** %HMWS in 10 mg/mL mAb1 in combination with different HPβCD concentrations |   **Figure S3.** %HMWS in 10 mg/mL mAb1 in combination with buffer, sugar, and interfacial stabilizer |
| --- | --- | --- |

|  | **Full chromatogram** | **Zoom to integrated HMWS** |
| --- | --- | --- |
| **t0** | 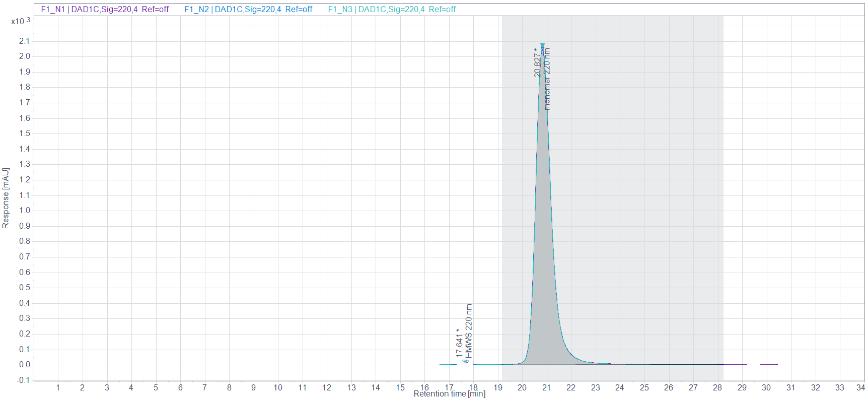 | 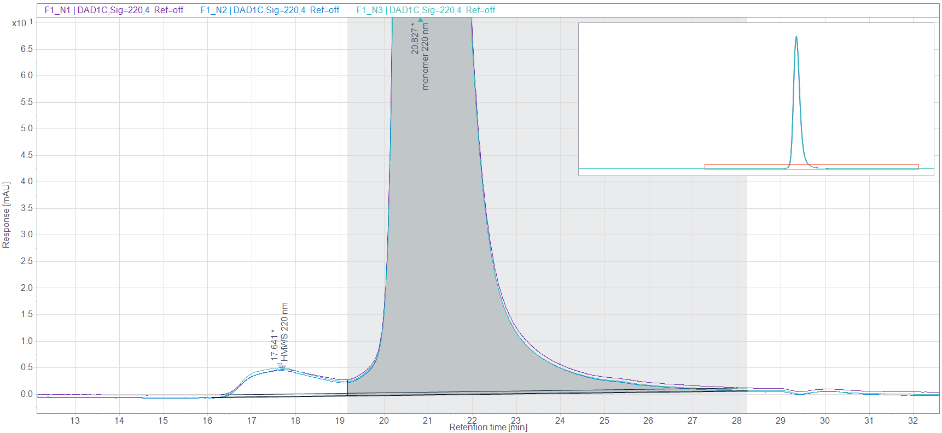 |
| **12 months** | 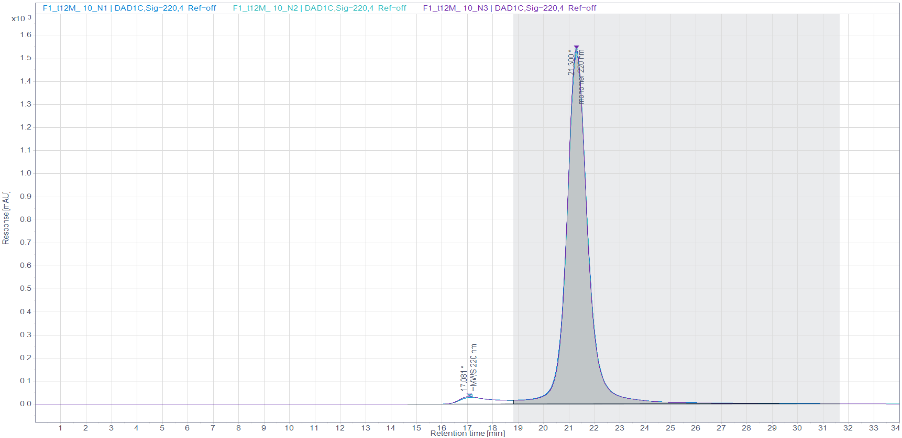 | 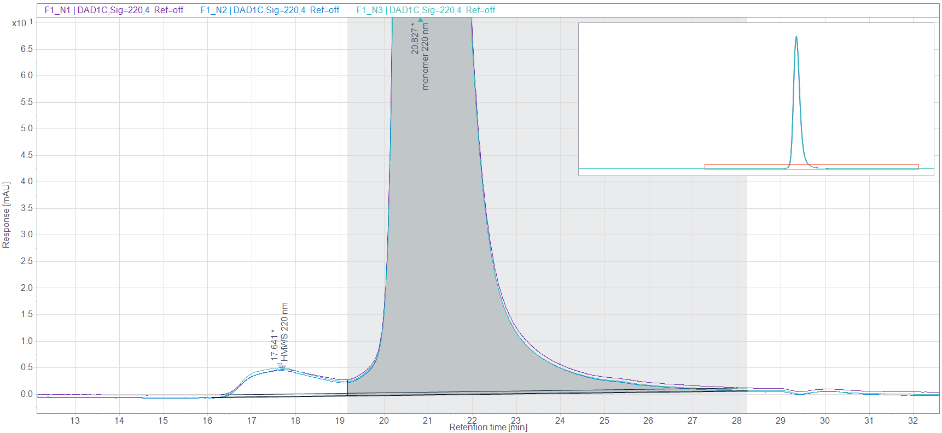 |

**Figure S4.** Exemplary chromatogram acquired by UV detection after HP-SEC of 10 mg/mL mAb 1, comparative for t0 and after 12-month storage

# High molecular weight species (HMWS) of mAbs in non-formulated and fully formulated DS measured by HP-SEC at t0 and after 12M at for -70 and -40 °C

**Table S1.** HMWS (%) for all tested mAbs in non-formulated (N) and fully formulated (F) for t0 and after 12 months at −70 °C, −40 °C.

| Formulation | | t0 | t12 | |
| --- | --- | --- | --- | --- |
|  |  |  | -70 °C | -40 °C |
| mAb 1 | N | 0.61 | 0.49 | 0.74 |
|  | F | 0.56 | 0.48 | 0.58 |
| mAb 2 | N | 2.91 | 2.63 | 3.05 |
|  | F | 2.88 | 2.86 | 2.92 |
| mAb 3 | N | 4.38 | 4.52 | 5.17 |
|  | F | 5.15 | 4.82 | 5.12 |
| mAb 4 | N | 1.29 | 1.55 | 1.90 |
|  | F | 1.36 | 1.51 | 1.48 |

# High molecular weight species (HMWS) of mAb 1 formulations measured by HP-SEC at t0 and after 12M at for -70, -40, and -10 °C

**Table S2.** HMWS (%) for mAb 1 across all tested formulations at t0 and after 12 months at −70 °C, −40 °C, and −10 °C.

| Excipients [mM] | | | | HMWS [%] | | | |
| --- | --- | --- | --- | --- | --- | --- | --- |
| Histidine | Sucrose | HPβCD | PS80 | t0 | t12 | | |
|  |  |  |  |  | -70 °C | -40 °C | -10 °C |
| - | - | - | - | 0.63 | 0.49 | 0.74 | 3.21 |
| - | 200 | - | - | 0.56 | 0.48 | 0.58 | 0.71 |
| - | 25 | - | - | 0.48 | 0.64 | 0.71 | 0.94 |
| - | 13 | - | - | 0.65 | 0.63 | 0.72 | 0.92 |
| - | 3 | - | - | 0.69 | 0.66 | 0.75 | 2.55 |
| - | 200 | 6.84 | - | 0.62 | 0.66 | 0.73 | 0.78 |
| - | - | 6.84 | - | 0.60 | 0.70 | 0.77 | 1.23 |
| - | - | 3.42 | - | 0.63 | 0.67 | 0.75 | 1.21 |
| - | - | 0.34 | - | 0.65 | 0.90 | 0.76 | 1.71 |
| - | 200 | - | 0.3 | 0.69 | 0.70 | 0.67 | 0.56 |
| 10 | 200 | 6.84 | - | 0.69 | 0.70 | 0.48 | 0.42 |
| 10 | 200 | - | - | 0.68 | 0.68 | 0.53 | 0.45 |
| 10 | - | - | - | 0.67 | 0.95 | 0.51 | 2.82 |
